# A Structured Management Approach to Implementation of Health Promotion Interventions in Head Start

**DOI:** 10.5888/pcd10.130015

**Published:** 2013-09-12

**Authors:** Ariella Herman, Bergen B. Nelson, Carol Teutsch, Paul J. Chung

**Affiliations:** Author Affiliations: Ariella Herman, Carol Teutsch, Johnson & Johnson Health Care Institute, Anderson School of Management, University of California, Los Angeles (UCLA); Paul J. Chung, Department of Pediatrics, David Geffen School of Medicine at UCLA, Children’s Discovery and Innovation Institute, Mattel Children’s Hospital UCLA, Department of Health Policy and Management, UCLA Fielding School of Public Health, Los Angeles, California, and RAND Health, The RAND Corporation, Santa Monica, California.

## Abstract

Improving the health and health literacy of low-income families is a national public health priority in the United States. The federal Head Start program provides a national infrastructure for implementation of health promotion interventions for young children and their families. The Health Care Institute (HCI) at the Anderson School of Management at the University of California, Los Angeles, developed a structured approach to health promotion training for Head Start grantees using business management principles. This article describes the HCI approach and provides examples of implemented programs and selected outcomes, including knowledge and behavior changes among Head Start staff and families. This prevention-focused training platform has reached 60,000 Head Start families in the United States since its inception in 2001. HCI has demonstrated consistent outcomes in diverse settings and cultures, suggesting both scalability and sustainability.

## Introduction

Compared with children from high-income families, children from low-income families in the United States face disproportionate health challenges and have worse reported child health status (1) and health risks, including higher than average rates of childhood obesity (2) and dental caries (3). Effective health promotion and disease prevention strategies have the potential to reduce the health burdens of vulnerable children (4,5). Families most vulnerable to child health risks may also have the lowest levels of literacy and health literacy (6). Recent federal policy initiatives have emphasized improving health literacy as a major avenue for reducing health disparities (7).

Head Start (HS), a national early childhood program for low-income families in the United States, was created in 1964 as part of the War on Poverty. HS and Early Head Start (EHS) programs annually serve approximately 1 million children aged 0 to 5 years. Recognizing the important relationship between health and school readiness, HS has long required its grantees to coordinate health-related services such as basic screenings, health education, and referrals to health providers (8). Each grantee has infrastructure to coordinate services as well as support for routine home visits and parent education workshops. A director and designated managers are typically provided in 6 service areas: education, health, mental health, nutrition, disabilities, and family services.

Since 1991, the Anderson School of Management at the University of California, Los Angeles (UCLA) has led annual leadership training for HS administrators. The UCLA/Johnson & Johnson Head Start Management Fellows Program has trained more than 1,400 HS leaders from grantee centers across the United States. On the basis of information provided via a 2001 survey of HS directors, the UCLA/Johnson & Johnson Health Care Institute (HCI) was formed to improve health literacy among HS families. The survey indicated that although grantees had access to health materials and resources to conduct health education trainings, these sessions were often poorly attended and the materials were not well understood (9).

HCI responded by developing a structured framework for health promotion that builds staff leadership capacity using systematic training and implementation strategies. Trained HS staff implement health promotion programs for their families using culturally adapted, low-literacy materials on various prevention topics. Family trainings include experiential group learning activities and hands-on skill building. We have found this approach to health promotion implementation to be a powerful way to motivate family participation and engagement; since 2001, HCI-trained staff from 240 HS grantees have reached 60,000 families across the United States.

The purpose of this article is to describe the unique, systematic process HCI uses to implement health promotion programs that consistently and successfully engage families to achieve measurable outcomes. We review selected results from national implementation efforts using this training approach and describe the potential of the model as a platform for ongoing health promotion for low-income families nationwide.

## Description of the HCI Approach

### Theoretical framework

Our approach to health promotion recognizes the multiple levels of influence on health behavior and outcomes, from organizational-level factors to social interactions within and among families in a community. At the onset of the training process, HS leaders identify local health education priorities and assess their organizational capacity for implementation. These leaders then train their staff using group structures to promote social interactions and learning among staff, which then diffuses to families through training events, home visits, and classroom activities. The ultimate goal is to affect individual-level health behaviors of parents and children. This multilevel approach reflects an integrated theoretical framework based on several established models of health promotion and behavior, including health promotion planning, social ecology, Life Course Health Development, and community partnering models, which have been described previously (10). This integrated theoretical framework has informed our practical approaches to implementation, designed to improve health behaviors using a structured process.

### Team formation and initial planning

**Figure 1:**
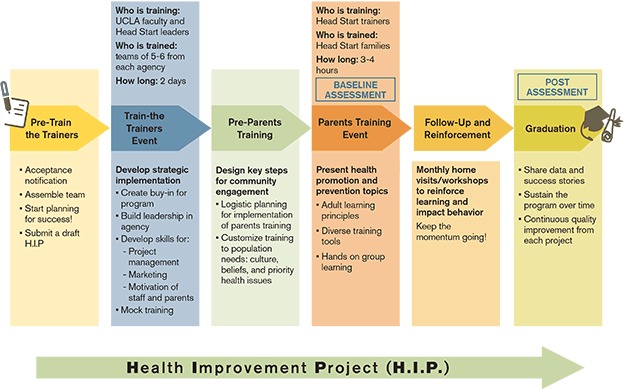
Health Care Institute strategic implementation.

The process HCI uses for health promotion training and implementation in HS begins with formation of a leadership team from each participating HS grantee, including the director and at least 2 others such as health managers, family service managers, and, whenever possible, a family from the program or a community partner (Figure 1). This defined team structure builds organizational investment and enhances the potential for diffusion and sustainability within each HS grantee, possibly in part by engaging early adopters within organizational social networks (11).

During the initial training process, HS leaders learn strategic management principles they can apply to health promotion program implementation. With guidance from HCI, each grantee team develops a strategic plan, the Health Improvement Project (HIP). The HIP is based on the Business Improvement Project, a tool used in the Management Development for Entrepreneurs executive training program at the UCLA Anderson School of Management (12). The HIP incorporates many elements of the previously established health planning model (13), including systematic articulation of goals, objectives, desired outcomes, organizational analysis, timelines, and evaluation plan. This tool builds leadership capacity for implementation of health promotion interventions as well as other projects.

### Training

After an initial planning meeting, leadership teams from as many as 50 HS grantees attend a 2-day train-the-trainer (TTT) event organized by HCI. Curriculum topics include strategic planning, project management, parent and staff motivation, marketing, and community relations. Teams use this time to refine their HIPs. A mock parent training demonstration during the TTT allows staff attendees to “walk in their families’ shoes” and learn the importance of audience engagement and interaction. In addition to the presentation of health education materials, the trainings include hands-on activities using topic-relevant tools (eg, thermometers, medication measuring devices, fruits and vegetables) and incentives such as door prizes. After the TTT, HS leadership teams return home to implement training events, customized to meet the needs of local communities, for staff and parents at their grantee sites.

The leadership teams train their entire HS staff using topic-specific health promotion materials provided by UCLA. HCI staff, faculty, and field-based partners provide technical assistance and advice throughout the implementation process. The staff training provides a model for the staff on how to lead interactive and engaging sessions for parents and children. Following the staff trainings, grantees engage their local communities, applying newly learned marketing strategies to engage local businesses, services, and health professionals and to secure in-kind donations and media coverage for the parent training event. At this point, grantees adapt their implementation strategies to meet local needs, translating materials into appropriate languages, incorporating cultural food preferences, and adding creative features to the event. The outreach process helps to build anticipation for the event and promote high attendance rates.

A key goal of logistics planning is reducing barriers to attendance by providing transportation, child care, and a complimentary healthful meal and holding trainings during hours that maximize parent availability (including weekends and evenings). Health materials developed by HCI are written at a low literacy level, using graphics and limited text, and generally include slides for trainers and books and handouts for parents to take home. The training blends many educational techniques: audio, visual, hands-on, group with peer-to-peer teaching, and handouts for home use. The event also provides the opportunity for social interaction and networking among parents.

Following the parents’ training, typically 3 home visits or workshops by HS staff are dedicated to reinforcement of the concepts learned at the initial training. These reinforcement activities help maintain family and staff engagement and focus on family assets that facilitate positive health behavior changes. Classroom teachers are encouraged to incorporate the health topics into daily classroom activities and curricula. Again, grantees have flexibility in designing the reinforcement activities that best meet local needs and cultures and use existing HS infrastructure, aligning activities with HS program performance standards.

At the conclusion of the reinforcement period, usually 3 to 4 months after the initial training, a graduation event celebrates families’ participation. This event is a forum for sharing success stories and building relationships. Certificates of completion enhance parents’ sense of accomplishment in building skills to care for their families.

### Measurement

For each implemented health promotion module HCI has collected preintervention and postintervention data from HS staff and parents. The baseline staff and parent measurements are generally done at the staff and parent training sessions and include written questionnaires to measure topic-specific health knowledge and self-reported behaviors. The same questionnaire is given again at the graduation session to measure changes in knowledge and behaviors. If literacy limitations pose problems for families to complete these written questionnaires independently, they are administered orally by staff.

## Selected Implementation Results

**Figure 2:**
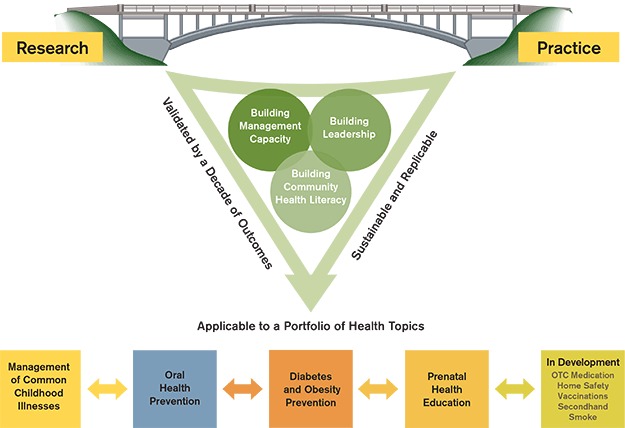
Structured approach to health promotion. Abbreviation: OTC, over the counter.

Once HS grantees participate in the initial TTT, they can implement other health promotion modules using a similar approach with new topic-specific materials introduced during webinars by HCI. From 2001 to 2012, we trained 240 HS grantees in 43 states to implement health promotion interventions on management of common childhood illnesses, oral health, and obesity prevention, reaching a total of 60,000 HS families. HCI materials have been translated into 7 languages and adapted for 10 ethnicities. Additional modules, such as use of over-the-counter medicines, home safety, vaccines, prenatal care, sun safety and secondhand smoke avoidance, are in various stages of development and testing (Figure 2).

The first topic HCI addressed in 2001 was management of common childhood illnesses, designed to help parents treat common symptoms at home and learn practical skills such as how to use a thermometer and measure over-the-counter medicines. Parents also learned how to use a simple health reference book and when to consult a health care provider. Comparison of data from pre- and postintervention parent questionnaires showed a significant decrease in reported number of missed school and work days and significant decreases in reported numbers of emergency department and doctor visits (14). HCI then developed an oral health module, including a resource information book (15), and has trained approximately 3,000 families on preventive oral health. This module was also adapted to an early childhood education setting outside of the United States and demonstrated positive pre- to postintervention behavior changes in oral hygiene practices and a 50% reduction in plaque index levels in a sample of 300 children (16).

Most recently, HCI has tried to address the national obesity epidemic with a 3-cohort ecologic model, “Eat Healthy, Stay Active!,” which trains staff, parents, and children to improve nutrition and increase physical activity. The national pilot study involved 6 HS grantees in 5 states and demonstrated significant reductions in body mass index (BMI) among parents, staff, and children, and a decrease in the proportion of obese children (30% to 21%, *P* < .001) and adults (45% to 40%, *P* < .001) over 6 months. We also documented increased nutrition knowledge, positive nutrition-related behavior changes, and increased frequency of physical activity (17). Not surprisingly, weight changes in parents were associated with weight changes in children, underscoring the importance of addressing the health of not only children but also those who teach children and establish cultural and social contexts for health behaviors across generations, consistent with the social-ecologic (18) and Life Course Health Development (19) models.

In addition to quantitative data, HCI has collected anecdotes suggesting that these health trainings can activate parents and capitalize on their intrinsic desire to meet their children’s health needs. Families report continued use of the health materials and increased attendance at other school events. Average parent attendance rates for HCI trainings are 85% nationally. We believe the approach facilitates engagement at many levels, building relationships between staff and parents and between HS and the local community. As one HS director noted, “The parents who are engaged in HCI trainings have been much more motivated to become involved in the program as a whole. We have seen increased participation in many of our Head Start parent training opportunities and other events as a direct result of their HCI participation.”

## Lessons Learned

Although the published findings described above have been replicated widely, we have rarely had a negative experience, which illustrates the importance of maintaining the integrity of the structured processes we have described. One grantee elected not to participate in any training events and simply distributed the HCI educational materials to staff and families. Measurements of pre- and postintervention knowledge and behavior for this grantee showed no change (M. King, oral communication, December 2011), suggesting that simply distributing written information is likely ineffective. A few grantees started the planning and training process but never completed implementation — these grantees tended to have directors who were not invested (ie, did not attend the TTT or left the grantee organization before local implementation), underscoring the importance of the grantee leadership teams as organizational drivers in the diffusion of new programs. Finally, barriers to parent participation are generally practical ones, such as lack of time, transportation, or child care, but may also include social factors, such as mistrust of staff because of prior negative experiences or perceived exclusion because of linguistic or cultural differences. Our training process encourages staff to recognize and address these barriers and to approach all families with the utmost respect, building on the trusting relationships usually established during the HS enrollment process.

## Discussion

HCI has applied a management training approach to health promotion implementation, using systematic strategies for planning, training, and engaging staff and families through a social-ecological framework. The HCI health promotion training model described in this article is a promising approach that has been replicated widely and disseminated across many HS sites, many of these implementing programs year after year. After attending just one TTT event, grantees are able to repeat the program and apply the same approach to multiple health topics. The HCI experience highlights the value of a systematic approach to dissemination of health promotion, consistent with dissemination frameworks for evidence-based health promotion practices reported previously (20). This approach to health promotion for low-income families represents a valuable and potentially cost-effective way to promote prevention and reduce health disparities in a vulnerable population.

The studies conducted by HCI to date are limited by lack of comparison groups, making the changes seen from pre- to postintervention measurement subject to selection bias. Future studies should measure the effectiveness of the HCI approach more rigorously using an experimental or quasiexperimental study design and also measure outcomes beyond the 6-month intervention period. Also, we have focused on measuring knowledge and behavior changes among families and staff but have limited implementation data. A more precise understanding of which program components are most important would be useful, as well as which organizational-level factors influence individual-level outcomes.

Another potential disadvantage of the HCI approach is that it is initially time-intensive, requiring multiple training sessions and reinforcement activities. We have found, however, that this investment of time and energy yields important intangible dividends, such as leadership capital and parent engagement. We believe this approach is essential to engage staff and families consistently and to promote positive behavior changes, and have found that efficiency increases with grantee expertise. Now, in partnership with the American Academy of Pediatrics as part of the Head Start National Center on Health, the HCI model is poised for wider dissemination in HS as well as across other early childhood programs nationwide.
